# Impact of a 12‐Element Nurse‐Led Tracheostomy Care Bundle on Stoma Integrity, Respiratory Distress and Suspected Pneumonia‐Related Outcomes Among Patients With Tracheostomies: A Quasi‐Experimental Study

**DOI:** 10.1002/nop2.70861

**Published:** 2026-09-30

**Authors:** Eman Yasser Hammouda, Mohammed Elsayed Zaky, Mona Saad Abdelwareth Ahmed, Walaa El‐Khanany Zahran, Hend El‐Sayed Mansour

**Affiliations:** ^1^ Faculty of Nursing, Critical Care and Emergency Nursing Mansoura University Mansoura Egypt; ^2^ Faculty of Nursing, Medical Surgical Nursing Department Cairo University Cairo Egypt; ^3^ Department of Medical and Surgical Nursing, College of Nursing Princess Nourah bint Abdulrahman University Riyadh Saudi Arabia; ^4^ Faculty of Nursing, Medical Surgical Nursing Department Damietta University Damietta City Egypt

**Keywords:** critically ill, pneumonia prevention, respiratory distress, stoma integrity, tracheostomy care bundle

## Abstract

**Aim:**

To evaluate the impact of a 12‐element nurse‐led tracheostomy care bundle, compared with routine care, on stoma integrity, respiratory distress, and pneumonia‐related outcomes among patients with tracheostomies.

**Design:**

A quasi‐experimental study was conducted among 80 critically ill patients with tracheostomies admitted to Mansoura University Hospital. Participants were allocated into a study group receiving a 12‐element nurse‐led tracheostomy care bundle and a control group receiving routine care (40 patients per group). Patients were followed for 15 days. Data were collected using stoma‐site assessment, the National Early Warning Score (NEWS), the Respiratory Distress Observation Scale (RDOS), and the modified Clinical Pulmonary Infection Score (CPIS). Statistical analyses included chi‐squared tests, Friedman tests for repeated measurements, and effect size estimation using partial eta squared.

**Results:**

Baseline demographic and clinical characteristics were comparable between groups (*p* > 0.05). Primary longitudinal repeated‐measures models revealed significant Group × Time interaction effects favouring the nurse‐led bundle for physiological stability (NEWS: *F* = 7.838, *p* < 0.001, partial eta squared = 0.086), stoma integrity composite scores (*F* = 5.849, *p* < 0.001, partial eta squared = 0.048), and respiratory distress trajectories (RDOS: *F* = 4.382, *p* = 0.014, partial eta squared = 0.036). Secondary day‐specific analyses showed lower stoma‐site infection signs (swelling, purulent discharge, skin breakdown, redness, warmth, and foul drainage) in the intervention cohort during follow‐up. Regarding pneumonia‐related outcomes, CPIS‐indicated suspected respiratory infection occurred less frequently in the study group (1‐week: 10.0% vs. 20.0%; 2‐week: 5.0% vs. 10.0%), though the difference was not statistically significant (Chi‐square = 3.710, *p* = 0.168).

**Conclusions:**

A 12‐element nurse‐led tracheostomy care bundle was associated with improved stoma integrity, reduced observed respiratory distress, and favourable changes in physiological stability among patients with tracheostomies. Although lower proportions of CPIS‐indicated suspected respiratory infection were observed in the bundle group, the difference was not statistically significant. Further multicentre controlled studies are required to determine the effect of standardized tracheostomy care bundles on pneumonia‐related outcomes.

**Implications for the Profession and Patient Care:**

Standardizing tracheostomy management via a nurse‐led bundle provides a structured, evidence‐informed framework for ICU practice. For nurses, it ensures consistent airway management, stoma assessment, suctioning, humidification, cuff pressure monitoring, and documentation, enhancing procedural fidelity and patient safety. For patients, bundle adherence supports stoma integrity, reduces respiratory distress, and promotes physiological stability. These improvements may optimize ICU workflows, guide timely interventions, and support care transitions such as decannulation and discharge. Implementation should include staff training, standardized checklists, and monitoring to ensure fidelity and adaptability. While preliminary results are promising, further evaluation in diverse clinical settings is recommended before broad adoption.

**Patient or Public Contribution:**

No patient or public contribution.

## 1 Introduction

Critically ill patients (CIPs) in intensive care units (ICUs) present complex clinical profiles requiring high‐acuity invasive procedures and continuous monitoring (Chen and Gong 2022). Tracheostomy is a cornerstone of airway management in this setting, facilitating prolonged mechanical ventilation, airway protection, and secretion clearance. Approximately 10%–15% of mechanically ventilated ICU patients require this intervention (Abe et al. 2018; Abril et al. 2021; Whitmore et al. 2020). In the Egyptian healthcare context, primary indications include prolonged endotracheal intubation (80.5%) and diaphragmatic paralysis (19.5%) (El‐Anwar et al. 2017).

## 2 Background

Tracheostomies carry inherent risks for airway obstruction, accidental decannulation, and stoma‐site infection (Alenezi et al. 2021; McMahon et al. 2023). These risks are not static; they arise directly from lapses in specific nursing actions inadequate suctioning permits secretion accumulation and airway obstruction, unmonitored cuff pressure predisposes to mucosal injury and leak‐related aspiration, inconsistent stoma assessment delays detection of local infection, and insufficient humidification promotes mucus plugging. Suboptimal management of these elements contributes to extended mechanical ventilation, prolonged ICU stay, and increased mortality (Rubin et al. 2020). Because each of these failure points is nursing‐sensitive, a structured, nurse‐led response rather than isolated corrective measures is required to interrupt this complication pathway (Sayed et al. 2026).

Patient safety is central to high‐quality intensive care, and nurses are positioned to lead this safety culture through consistent training and adherence to clinical bundles (Patil et al. 2023). Structured tracheostomy care bundles have been associated with reduced practice inconsistency (Hall et al. 2017), and standardized protocols and educational interventions have been linked to improvements in fragmented care delivery and nursing performance (Allam et al. 2024; Lima et al. 2024).

A tracheostomy care bundle consists of a cohesive set of routine practices designed to maintain the integrity of the tube flange, inner cannula, and stoma site (Carter and Notter 2023). The 12‐element bundle applied in this study operationalizes this through continuous physiological surveillance, scheduled suctioning, humidification, cuff pressure monitoring, structured stoma assessment, oral care, tube security checks, emergency equipment readiness, resuscitation preparedness, mobilization, and standardized documentation with defined escalation criteria for clinical deterioration. Each element targets a specific mechanism of harm rather than functioning as a generic checklist. Adherence to bundled tracheostomy care has been associated with shorter decannulation time and reduced ICU length of stay, mortality, and respiratory distress in prior work (Allam et al. 2024; Sayed et al. 2026; Suliman et al. 2024).

Despite this evidence base, three distinct gaps remain unaddressed in the Egyptian critical care context. First, there is local practice variation: cuff pressure monitoring and infection‐prevention steps are applied inconsistently across units in the absence of national guidelines (Khanum et al. 2022; Suliman et al. 2024). Second, nurse‐led as opposed to physician‐led or multidisciplinary tracheostomy bundles remain largely unstandardized in Egyptian ICUs, with no locally validated protocol specifying nursing scope and sequencing (Abdulrahman et al. 2021). Third, existing regional studies of tracheostomy care bundles have evaluated process adherence or staff knowledge rather than direct, longitudinally measured patient outcomes such as stoma integrity, respiratory distress trajectory, and pneumonia incidence. This study addresses these three gaps by evaluating the implementation of a standardized, 12‐element nurse‐led tracheostomy care bundle against these specific outcome measures in the Egyptian ICU setting.

### 2.1 Hypotheses

Hypothesis 1 *Patients managed with the nurse‐led tracheostomy care bundle will demonstrate significantly better stoma integrity, reflected in a lower incidence of local stoma‐site infection signs, than patients receiving routine care*.

Hypothesis 2 *Patients managed with the nurse‐led tracheostomy care bundle will demonstrate a significantly lower frequency of respiratory distress, as measured by the RDOS, than patients receiving routine care*.

Hypothesis 3 *Patients managed with the nurse‐led tracheostomy care bundle will demonstrate a significantly lower incidence of CPIS‐indicated (suspected) pneumonia than patients receiving routine care*.

### 2.2 Aim and Objectives

To evaluate the effectiveness of a 12‐element nurse‐led tracheostomy care bundle, compared with routine care, on stoma integrity, respiratory distress, physiological stability, and CPIS‐indicated pneumonia incidence among CIPs with tracheostomies.

## 3 Methods

### 3.1 Design

A quasi‐experimental research design was utilized in this study that adhered to TRENDS, allowing the assessment of intervention effects in target populations without randomization. This design is especially well‐suited for clinical nursing research in real‐world healthcare environments (Polit and Beck 2019).

### 3.2 Setting and Sample

This quasi‐experimental study was conducted at Mansoura University Hospital, Egypt, involving critically ill adult patients with tracheostomies admitted to the Anaesthesia, Surgical, and Pain Management ICU, with subsequent follow‐up in the Ear, Nose, and Throat (ENT) Department when applicable. The ICU served as the primary recruitment and intervention site, where eligible patients received either the nurse‐led 12‐element tracheostomy care bundle or routine tracheostomy care according to their assigned group. The ENT Department participated in follow‐up assessments for patients transferred after stabilization or ICU discharge while continuing tracheostomy management. Both departments are tertiary‐care units equipped with advanced monitoring facilities and staffed by trained healthcare professionals. The nurse‐to‐patient ratio was approximately 1:2 in the ICU and 1:3 in the ENT Department.

A consecutive sampling approach was used to recruit adult patients undergoing elective or emergency tracheostomy during the study period. Group allocation was governed strictly by a non‐randomized, sequential temporal cohort design to prevent inter‐group contamination during staff educational implementation. All consecutive eligible patients admitted to the unit between January and April 2025 were allocated to the control cohort (routine care). Following a 4‐week nurse educational training and protocol standardization period in May 2025, all consecutive eligible patients admitted between June and September 2025 were allocated to the intervention cohort (12‐element bundle). Individual participant allocation was determined exclusively by admission date to reduce clinician selection bias during assignment.

### 3.3 Sample Size and Power

The required sample size was estimated using G*Power software (version 3.1.9.7). The calculation was based on a moderate expected effect size, a significance level of α = 0.05, statistical power of 80% (1 − *β* = 0.80), and a 1:1 group allocation ratio, resulting in a minimum required sample of 80 participants (40 patients per group). Although repeated measurements of clinical outcomes (NEWS and RDOS) were collected longitudinally, the initial sample estimation was based on between‐group comparisons because the primary analysis plan included categorical outcome comparisons.

### 3.4 Inclusion and Exclusion Criteria

Eligible participants were adults aged ≥ 18 years who underwent elective or emergency tracheostomy and were admitted to the selected ICUs during the study period. Patients were excluded if they had pre‐existing pneumonia, evidence of stoma‐site infection at enrollment, or any condition that prevented standardized assessment of tracheostomy‐related outcomes. Chronic diseases and other comorbidities were not considered exclusion criteria because they represent common clinical characteristics among CIPs requiring tracheostomy. Instead, comorbidities were recorded as baseline clinical characteristics and compared between groups to assess baseline comparability and identify potential confounding factors.

This study used a quasi‐experimental design; therefore, participants were not randomly allocated to study groups. Group assignment followed the clinical implementation pathway of the tracheostomy care bundle within the study setting. Patients receiving routine tracheostomy care were included in the control group, whereas patients receiving the nurse‐led 12‐element tracheostomy care bundle were included in the intervention group. This pragmatic allocation reflected the implementation of the intervention within routine clinical practice; however, the absence of random allocation was recognized as a potential source of selection bias and was considered when interpreting the findings.

Eligible patients were recruited consecutively according to the predefined inclusion and exclusion criteria. Recruitment continued until the planned evaluable sample size of 40 participants in each group was achieved. Participants were followed daily for a maximum of 15 consecutive days after tracheostomy insertion or until hospital discharge, whichever occurred first. The 15‐day follow‐up period was selected based on the longest post‐tracheostomy observation period available during the study timeframe.

During follow‐up, six enrolled participants did not complete the predefined observation period. Two participants from the intervention group were transferred to another facility, and four participants from the control group died from causes considered unrelated to tracheostomy management. Because these participants did not complete the predefined follow‐up assessments, they were not included in the evaluable cohort analysis. Replacement participants were subsequently recruited consecutively using the same eligibility criteria and recruitment procedures to achieve the planned evaluable sample size. No participant was replaced after completing intervention exposure or outcome assessment.

AS shown in Figure 1, a total of 94 participants were assessed for eligibility, of whom 14 were excluded based on predefined criteria (pre‐existing pneumonia, *n* = 6; pre‐existing stoma infection signs, *n* = 3; legal guardian declined consent, *n* = 5). Initially, 80 participants were enrolled and allocated to the study cohorts. During follow‐up, six participants did not complete the predefined observation period (four deaths in the control group unrelated to tracheostomy care and two transfers in the intervention group to another facility). To maintain statistical power, six replacement participants were consecutively recruited using identical eligibility criteria, resulting in a total of 86 patients enrolled across the study period to yield the final evaluable analysis population of 80 participants (40 in the control group and 40 in the intervention group).

**FIGURE 1:**
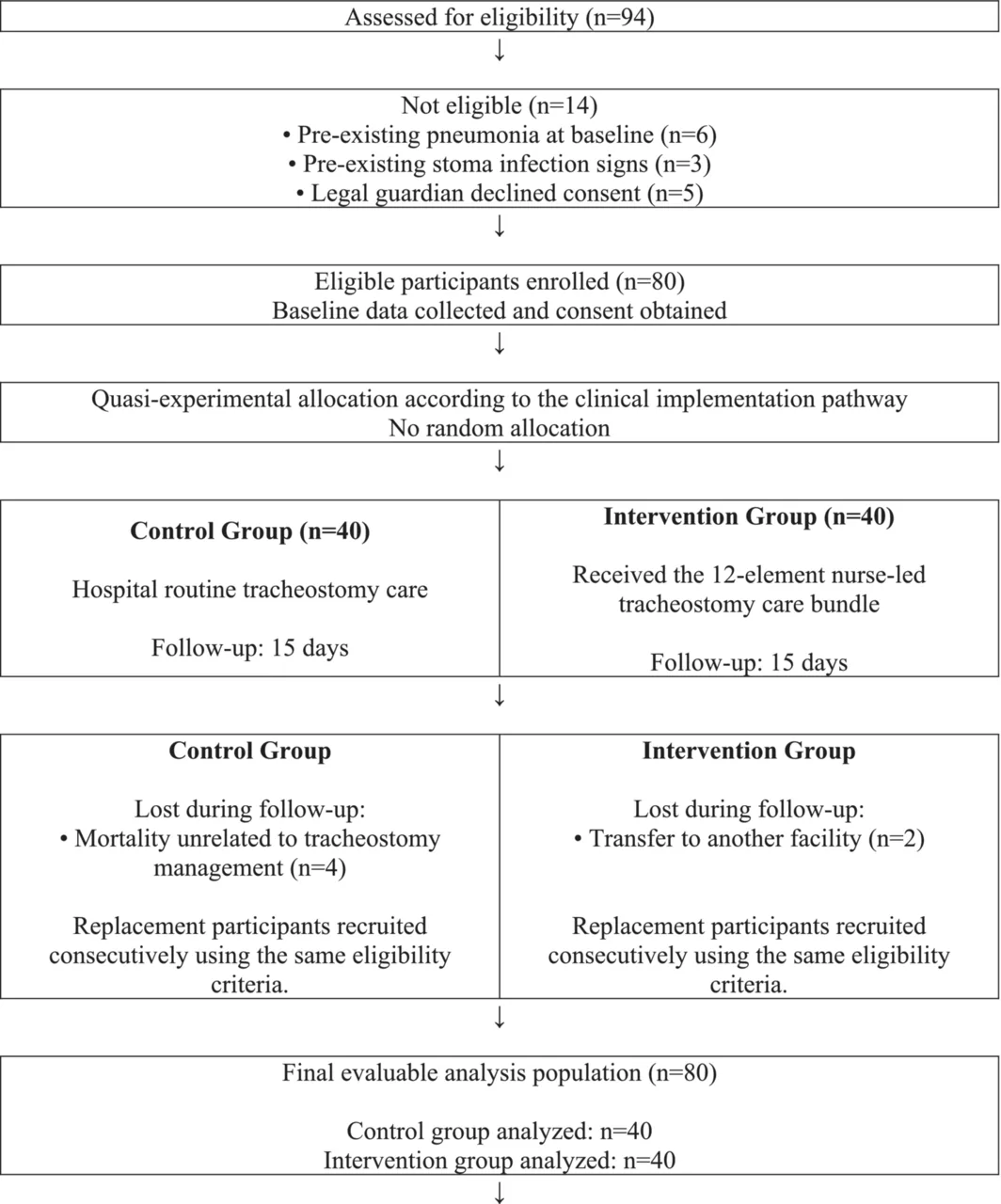
Flowchart of participants' recruitment, allocation, intervention, follow‐up, and analysis.

### 3.5 Data Collection Tools and Methods

Four tools were utilized to collect data prior to the study.

#### 3.5.1 Part I: Demographic and Clinical Characteristics Sheet

A structured demographic and clinical characteristics sheet was developed based on a review of relevant literature regarding tracheostomy management in CIPs (Mussa et al. 2021; Mohmed Mahmod Allam et al. 2024). The tool was used to establish baseline comparability between groups and identify potential confounding factors. It consisted of two sections. The first section collected demographic characteristics, including age, gender, and smoking status. The second section assessed clinical characteristics, including ICU admission diagnosis, previous ICU admission, comorbidities, mechanical ventilation status, and tracheostomy‐related characteristics such as indication and type of tracheostomy.

#### 3.5.2 Part II: National Early Warning Score

The National Early Warning Score (NEWS), developed by Williams et al. (2012) was used to assess physiological status and detect clinical deterioration among patients with tracheostomies. NEWS integrates seven physiological parameters: respiratory rate, oxygen saturation, supplemental oxygen requirement, temperature, systolic blood pressure, heart rate, and level of consciousness (Burgos‐Esteban et al. 2022). Each parameter is scored from 0 to 3 according to the degree of physiological abnormality, with higher total scores indicating greater physiological disturbance.

NEWS scores were interpreted according to established risk categories: low clinical risk (NEWS 0–4), medium clinical risk (NEWS 5–6), and high clinical risk (NEWS ≥ 7). These categories were used to describe the degree of physiological deterioration and to guide clinical observation and escalation of care in accordance with institutional protocols.

NEWS was assessed daily from baseline until Day 15 or patient discharge, whichever occurred first, to provide ongoing surveillance of physiological deterioration throughout the post‐tracheostomy period. For patients discharged before Day 15, discharge marked the end of follow‐up because no further assessments were applicable. Discharge was recorded as a follow‐up endpoint rather than a NEWS category, and NEWS assessment was discontinued thereafter. The primary NEWS outcome was the change in physiological status over time, assessed through longitudinal changes in total NEWS scores and the distribution of patients across the predefined risk categories between the intervention and control groups.

#### 3.5.3 Part III: Respiratory Distress Observation Scale

The Respiratory Distress Observation Scale (RDOS), developed by Campbell (2008), was used to objectively assess respiratory distress in CIPs who may be unable to reliably communicate symptoms of dyspnea due to mechanical ventilation, sedation, altered consciousness, or other critical illness‐related factors. This characteristic makes RDOS particularly suitable for patients with tracheostomies in the intensive care setting, where respiratory discomfort may be reflected through observable physiological and behavioural manifestations. The RDOS consists of eight observable indicators: heart rate, respiratory rate, restlessness, paradoxical breathing, accessory muscle use, grunting, nasal flaring, and facial expression of fear. Each indicator is scored from 0 to 2, resulting in a total score ranging from 0 to 16, with higher scores indicating greater severity of respiratory distress.

RDOS assessments were performed at three predefined time points: immediately after tracheostomy insertion (baseline), postoperative Day 7, and postoperative Day 15 or at patient discharge, whichever occurred first. The baseline assessment was performed to establish the initial respiratory distress status before exposure to the assigned tracheostomy care approach. The Day 7 assessment was selected to evaluate early changes following continued tracheostomy management, whereas the final assessment represented the later respiratory status during follow‐up or the clinical status at discharge when applicable.

In this study, RDOS was evaluated as a repeated outcome measure to assess changes in respiratory distress severity over time between the intervention and control groups. RDOS was analysed both as a continuous score and as a categorical variable according to clinically observed severity levels (none, mild, moderate, and severe respiratory distress). The outcome of interest was the difference in respiratory distress trajectory over follow‐up between patients receiving the nurse‐led tracheostomy care bundle and those receiving routine care.

RDOS was assessed at predefined intervals rather than daily because it was intended as a standardized outcome measure for evaluating respiratory distress progression rather than a continuous physiological monitoring tool. Any acute clinical deterioration occurring between RDOS assessments was monitored through routine ICU observation, daily NEWS evaluation, and standard clinical assessment procedures. Clinical management decisions were performed according to the established ICU protocols and were independent of the scheduled RDOS outcome assessments.

#### 3.5.4 Part IV: Modified Clinical Pulmonary Infection Score

The modified Clinical Pulmonary Infection Score (CPIS) was used as a clinical screening tool to assess the probability of respiratory infection rather than to establish a definitive microbiological diagnosis of pneumonia, as microbiological confirmation was not consistently available during the study period. The original CPIS, developed by Pugin et al. (1991), includes six components: body temperature, white blood cell count, tracheal secretion characteristics, oxygenation status (PaO_2_/FiO_2_ ratio), chest radiographic findings, and microbiological culture results, with each component scored from 0 to 2. Because routine tracheal aspirate culture results required 24–48 h and were not consistently available for serial assessment, a modified five‐component CPIS excluding microbiological culture was used, as previously described by Chen et al. (2018) and Moradi Moghaddam et al. (2019). A CPIS score > 5 was considered suggestive of suspected respiratory infection.

CPIS was assessed at three predefined time points: immediately after tracheostomy insertion (baseline), on Day 7, and on Day 15 or at patient discharge, whichever occurred first. Baseline assessment established the patient's initial pulmonary status before implementation of the intervention. The Day 7 assessment was performed to identify early‐onset suspected respiratory infection during the early postoperative period, whereas the final assessment evaluated late‐onset suspected respiratory infection following prolonged tracheostomy care (Langer et al. 1987). Accordingly, CPIS findings were interpreted as indicators of CPIS‐indicated suspected respiratory infection rather than microbiologically confirmed pneumonia.

### 3.6 Composite Definition of Stoma Integrity Outcome

Stoma integrity was assessed by examining the tracheostomy site for local abnormalities, including redness, swelling, warmth, purulent or foul‐smelling drainage, and skin breakdown or ulceration. Each finding was documented separately, and the overall stoma‐site status was classified according to the presence or absence of any clinically significant abnormality.

Assessments were performed at three predefined time points: immediately after tracheostomy insertion (baseline), on postoperative Day 7, and on Day 15 or at patient discharge, whichever occurred first. The baseline assessment documented the initial condition of the stoma before intervention. Day 7 represented the early wound‐healing period, allowing detection of emerging local complications, whereas the final assessment evaluated the subsequent healing progress and persistence or resolution of local abnormalities. This follow‐up schedule was selected in accordance with the expected early healing period of the tracheostomy site (Ministry of Health, Oman 2025). A lower occurrence and severity of stoma‐site abnormalities indicated better stoma integrity.

### 3.7 Validity and Reliability

Content validity was assessed through expert review by a panel of five experts specialized in Medical‐Surgical Nursing and Critical Care Nursing. The experts independently evaluated the demographic and clinical characteristics sheet, National Early Warning Score (NEWS), Respiratory Distress Observation Scale (RDOS), and modified Clinical Pulmonary Infection Score (CPIS) regarding relevance, clarity, comprehensiveness, and applicability to CIPs with tracheostomies. Feedback and recommendations from the experts were reviewed by the research team, and necessary modifications were implemented accordingly.

The selection of NEWS and RDOS was based on their established clinical utility in monitoring physiological deterioration and assessing respiratory distress in acute care populations (Gerry et al. 2020; Campbell et al. 2010). Previous psychometric studies provide supporting evidence for the use of these instruments; however, they were considered external validation evidence and were not interpreted as confirmation of validity within the current study sample. The modified CPIS was used to assess CPIS‐indicated suspected respiratory infection based on available clinical parameters. Because microbiological data were not included, CPIS findings were not considered diagnostic confirmation of pneumonia and were interpreted cautiously as pneumonia‐related clinical outcomes.

For cultural adaptation, a forward‐backward translation procedure was conducted. The instruments were initially translated from English into Arabic by a bilingual healthcare professional familiar with clinical terminology. The translated versions were then independently back‐translated into English by a second bilingual reviewer. Discrepancies between the original and translated versions were reviewed and resolved by the research team to ensure conceptual equivalence. Face validity and feasibility of the translated tools and data collection procedures were evaluated during a pilot study involving eight patients, who were excluded from the final analysis.

Inter‐rater reliability was assessed during the pilot phase through independent paired assessments performed by the primary researcher and an independent critical care nurse assessor. Twenty paired observations were conducted using the NEWS, RDOS, and modified CPIS tools. Reliability was evaluated using the Intraclass Correlation Coefficient (ICC), demonstrating good agreement between raters, with ICC values of 0.82 for NEWS, 0.88 for RDOS, and 0.87 for modified CPIS. These findings indicate acceptable consistency of measurements between observers within the study assessment process.

Data collection was conducted from January to September 2025 through a structured sequential process consisting of four phases: (I) ethical approval, pilot testing, and study preparation; (II) participant recruitment, group allocation, and baseline assessment; (III) nurse training and implementation of the tracheostomy care bundle; and (IV) follow‐up assessment, outcome evaluation, and monitoring of intervention fidelity and safety outcomes.

### 3.8 Study Phases and Procedures

#### 3.8.1 Phase I: Ethical Approval, Pilot Study, and Study Preparation

Ethical approval was obtained from the institutional Research Ethics Committee and hospital administration before initiation of the study. The study procedures were conducted according to applicable ethical principles and institutional regulations. Written informed consent was obtained from patients' legal guardians because the majority of eligible patients were unable to provide informed consent independently due to their critical illness, mechanical ventilation, sedation, or altered consciousness. Guardians were informed about the study objectives, procedures, potential benefits and risks, voluntary participation, confidentiality measures, and the right to withdraw without affecting the received medical care. When participants regained decision‐making capacity during follow‐up, consent was reconfirmed whenever applicable according to institutional ethical requirements.

A pilot study was conducted on eight patients (10% of the calculated sample size), who were excluded from the final analysis. The pilot study aimed to evaluate the feasibility of recruitment procedures, data collection methods, documentation processes, and application of the study tools. Minor modifications were made to the demographic and clinical data collection sheet based on pilot findings. Reliability assessment of the study tools was performed during this phase through inter‐rater evaluation.

Before the beginning of the main study, operational definitions and scoring criteria for all assessment tools, including NEWS, RDOS, modified CPIS, and stoma‐integrity assessment, were standardized. Participating critical care nurses were oriented regarding assessment procedures and documentation requirements to ensure consistency throughout the study period.

#### 3.8.2 Phase II: Participant Eligibility, Recruitment, and Group Allocation

Eligible participants were adults aged ≥ 18 years who underwent elective or emergency tracheostomy and were admitted to the selected ICUs during the study period. Patients were excluded if they had: pre‐existing pneumonia at enrollment; evidence of stoma‐site infection at baseline; any condition preventing standardized assessment of tracheostomy‐related outcomes. The presence of chronic diseases or comorbidities was not considered an exclusion criterion because such conditions are common among CIPs requiring tracheostomy. Instead, comorbidities were recorded as baseline clinical characteristics and compared between groups to evaluate baseline comparability and potential confounding factors.

Because this study used a quasi‐experimental design, participants were not randomly allocated. Allocation was determined according to the sequential clinical implementation pathway of the tracheostomy care bundle within the study setting. During the initial study period (January–April 2025), eligible patients were consecutively recruited into the control group and received routine tracheostomy care according to the existing institutional protocol. Following completion of control‐group recruitment and implementation of the intervention protocol, a subsequent cohort of eligible patients admitted during the intervention period (June–September 2025) was consecutively recruited into the intervention group.

#### 3.8.3 Phase III: Baseline Assessment and Routine Care (Control Group)

Before initiation of routine care, baseline demographic and clinical characteristics were collected using Tool I. Baseline physiological stability, respiratory distress, suspected respiratory infection, and stoma condition were assessed using NEWS, RDOS, modified CPIS and stoma‐integrity assessment criteria, respectively.

Participants in the control group received routine tracheostomy care according to the institutional protocol, including: airway suctioning when clinically indicated; humidification according to unit practice; routine tracheostomy dressing changes; monitoring of tube position and patency; standard infection prevention measures. Outcome assessments were performed using the predefined assessment schedule applied throughout the study.

#### 3.8.4 Phase IV: Nurse Training and Intervention Implementation

After completion of control‐group recruitment, the principal investigator conducted a four‐week educational training programme for critical care nurses participating in the intervention phase in May 2025. The training focused on the scientific rationale of structured tracheostomy care, the clinical importance of standardized airway management, prevention of stoma‐related complications, infection prevention practices, and the correct application of each component of the care bundle.

The 12‐element nurse‐led tracheostomy care bundle was adapted from the ACI–ICCMU evidence‐based guideline (ACI‐ICCMU Tracheostomy Expert Group 2013) and included evidence‐based nursing interventions targeting airway management, tracheostomy stoma care, and infection prevention. Following completion of the training programme, eligible patients were consecutively recruited into the intervention group. Baseline demographic and clinical assessments were performed using the same procedures applied for the control group to ensure comparability between groups.

The tracheostomy care bundle was initiated within 24 h after tracheostomy insertion following completion of the baseline assessment and was continued for a maximum of 15 consecutive days or until patient discharge, whichever occurred first. The intervention was delivered by trained critical care nurses under the supervision of the principal investigator. Continuous guidance, feedback, and monitoring were provided throughout the intervention period to promote consistent implementation of all bundle components.

#### 3.8.5 Phase V: Follow‐Up, Outcome Assessment, and Intervention Fidelity

All participants were followed for a maximum of 15 consecutive days after tracheostomy insertion or until discharge, whichever occurred first. The same standardized outcome assessment procedures were applied to both groups using NEWS for physiological stability monitoring, RDOS for respiratory distress assessment, modified CPIS for suspected respiratory infection assessment, and stoma‐integrity assessment for evaluation of local tracheostomy‐site outcomes.

Whenever possible, outcome assessments were performed by a critical care nurse who was not directly involved in intervention delivery to minimize assessment bias. A standardized follow‐up sheet was maintained for each participant to document clinical observations, NEWS findings, and scheduled outcome assessments. Data collection procedures, operational definitions, and scoring criteria were standardized and applied equally in both groups. The researcher regularly reviewed completed records to ensure data completeness, accuracy, and consistency.

Intervention fidelity was maintained through the predefined 12‐element tracheostomy care bundle. Participating nurses received standardized training regarding all bundle components before intervention initiation, and adherence to the specified bundle elements was monitored throughout the intervention period to ensure consistent implementation.

#### 3.8.6 Phase VI: Participant Retention, Replacement, and Safety Monitoring

Participants who did not complete the predefined follow‐up period were documented within the participant flow process. During follow‐up, six participants were lost: two participants from the intervention group were transferred to another facility, and four participants from the control group died from causes considered unrelated to tracheostomy management.

Because these participants did not complete the predefined outcome assessments, they were not included in the final evaluable cohort. Replacement participants were consecutively recruited using the same eligibility criteria and recruitment procedures to achieve the predetermined evaluable sample size.

A total of 86 participants were enrolled to obtain the final evaluable cohort of 80 participants, including 40 participants in the intervention group and 40 participants in the control group. The participant flow diagram presents eligibility screening, group allocation, losses to follow‐up, replacement participants, and the final analysis populations.

Adverse events potentially related to the intervention were monitored throughout the follow‐up period. No adverse events directly attributable to implementation of the tracheostomy care bundle were observed.

### 3.9 Data Analysis

Data were coded and analysed using IBM SPSS Statistics version 26.0. Qualitative variables were presented as frequencies and percentages, and quantitative variables as mean ± SD according to data distribution. Baseline characteristics were compared using chi‐squared test for categorical variables and independent‐samples *t*‐test for continuous variables. Fisher's exact test or Monte Carlo two‐sided significance values were used when Chi‐square assumptions were not met.

The longitudinal change in NEWS was analysed using a repeated‐measures model accounting for within‐participant correlation. The model included group, time, and Group × Time interaction, with the interaction considered the primary test for differences in NEWS trajectories between groups. Estimates, 95% confidence intervals, *F* values, *p* values, and partial eta squared (*ηp* ^2^) were reported. Day‐specific NEWS category comparisons were performed as secondary analyses using chi‐square, Fisher's exact, or Monte Carlo tests as appropriate.

Within‐group changes in ordinal outcomes (RDOS, stoma‐integrity outcomes, and modified CPIS) were assessed using the Friedman test. Effect estimates with 95% confidence intervals were reported where applicable. Partial eta squared was only applied to the repeated‐measures model and not to Friedman analyses. A two‐sided *p* ≤ 0.05 was considered statistically significant.

### 3.10 Ethical Considerations

Ethical approval was obtained from the Research Ethics Committee of the Faculty of Nursing at Faculty of Nursing Mansoura University (Approval date: 1/9/2024—no. 0631), and administrative approval was obtained from the hospital authority. Written informed consent was obtained from participants or their legally authorized representatives before enrollment. For patients unable to provide consent due to their clinical condition, consent was obtained from legally authorized representatives, and consent was reconfirmed from participants who regained decision‐making capacity whenever applicable according to institutional requirements. Participation was voluntary, and withdrawal was permitted at any time without affecting medical care. All collected data were kept confidential and used exclusively for research purposes.

## 4 Results

Table 1 presents the demographic and clinical characteristics of the enrolled participants. No statistically significant differences were observed between the control and intervention groups regarding age distribution, sex, smoking status, admission diagnosis, previous ICU admission, comorbidities, mechanical ventilation status, or type of tracheostomy (*p* > 0.05 for all comparisons). The majority of participants were aged 40–49 years in both groups, with males representing 55.0% of the control group and 60.0% of the intervention group. Most participants had at least one comorbidity (90.0% in the control group and 92.5% in the intervention group), and mechanical ventilation was required in the majority of patients in both groups (87.5% and 90.0%, respectively). The distribution of admission diagnoses and tracheostomy types was also comparable between groups.

**TABLE 1:** Part I: patients' characteristics and health relevant data (*n* = 80).

Patients' characteristics	Control (*n* = 40), *n* (%)	Study (*n* = 40), *n* (%)	*χ* ^2^	*p*
Age				
20–29	3^a^ (7.5)	2^a^ (5.0)		
30–39	8^a^ (20.0)	10^a^ (25.0)		
40–49	12^a^ (30.0)	11^a^ (27.5)	0.938	0.919
50–59	8^a^ (20.0)	10^a^ (25.0)		
≥ 60	9^a^ (22.5)	7^a^ (17.5)		
Gender				
Male	22^a^ (55.0)	24^a^ (60.0)		
Female	18^a^ (45.0)	16^a^ (40.0)	0.205	0.651
Smoking status				
Current smoker	10^a^ (25.0)	3^b^ (7.5)		
Ex‐smoker	14^a^ (35.0)	21^a^ (52.5)	5.169	0.075
Non‐smoker	16^a^ (40.0)	16^a^ (40.0)		
Admission diagnosis				
Stroke	4^a^ (10.0)	8^a^ (20.0)		
Traumatic brain injury	3^a^ (7.5)	2^a^ (5.0)		
Myasthenia gravis	5^a^ (12.5)	2^a^ (5.0)		
Anaplastic carcinoma	8^a^ (20.0)	4^a^ (10.0)	4.868	0.444
Laryngeal tumour	8^a^ (20.0)	12^a^ (30.0)		
Lymphadenopathy	12^a^ (30.0)	12^a^ (30.0)		
Previous ICU admission				
Yes	22^a^ (55.0)	21^a^ (52.5)	0.050	0.823
No	18^a^ (45.0)	19^a^ (47.5)		
Comorbidity				
No	4^a^ (10.0)	3^a^ (7.5)		
Yes	36^a^ (90.0)	37^a^ (92.5)	0.157	0.692
If yes	*N* = 36	*N* = 37		
Cardiovascular disorders	16^a^ (44.4)	12^a^ (32.4)	1.113	0.291
Respiratory disease	10^a^ (27.8)	9^a^ (24.3)	0.113	0.737
Neurological diseases	3^a^ (8.3)	4^a^ (10.8)	0.129	0.719
GIT diseases	7^a^ (19.4)	13^a^ (35.1)	2.258	0.133
Renal diseases	2^a^ (5.6)	4^a^ (10.8)	0.668	0.414
Endocrinal diseases	12^a^ (33.3)	10^a^ (27.0)	0.345	0.557
Patient on MV				
Yes	35^a^ (87.5)	36^a^ (90.0)	0.125	0.723
No	5^a^ (12.5)	4^a^ (10.0)		
Type of tracheostomy				
Emergency	9^a^ (22.5)	12^a^ (30.0)		
Elective	31^a^ (77.5)	28^a^ (70.0)	0.581	0.446

*Note:* a,b: Bonferroni‐adjusted pairwise comparisons; values sharing the same superscript letter are not significantly different, whereas values with different superscript letters indicate significant differences. *p* ≤ 0.05 was considered statistically significant.

Abbreviations: *χ* ^2^, chi‐squared test; MC, Monte Carlo significance (two‐sided).

Table 2 shows that NEWS scores were analysed using a longitudinal repeated‐measures model accounting for within‐participant correlations across follow‐up. The model assessed the main effects of study group and time, as well as the Group × Time interaction. A significant Group × Time interaction indicates that the pattern of change in NEWS scores over time differed significantly between the control and study groups. Results are presented as model estimates with 95% confidence intervals (CIs), *F* statistics, *p* values, and partial eta squared (*ηp* ^2^) as an effect‐size measure. A *p* < 0.05 was considered statistically significant.

**TABLE 2:** Longitudinal changes in news risk categories and follow‐up status in the control and study group.

Parameter NEWS	Estimate	95% CI	*F*	*p*	Partial *η* ^2^
Group					
Control	2.15	2.07–2.22	5.841*	0.016*	0.005
Study	2.27	2.20–2.34			
Time	1.200	0.639–1.761	9.766*	< 0.001*	0.105
Group × Time	2.207	2.155–2.260	7.838*	< 0.001*	0.086

*Note:* Group × Time interaction represents the primary test of whether changes in NEWS trajectories differed between the control and intervention groups over the follow‐up period. Partial eta squared (*ηp* ^2^) was reported as the effect size for the repeated‐measures longitudinal model. A *p* ≤ 0.05 was considered statistically significant.

Abbreviations: CI, confidence interval; *F*, *F* statistic.

* Statistically significant at *p* ≤ 0.01.

Table 3 presents the daily distribution of National Early Warning Score (NEWS) risk categories among active inpatients across the 15‐day follow‐up period. Baseline physiological risk categories assessed on the first postoperative day were comparable between the control and intervention groups, showing no statistically significant difference (chi‐square = 1.892, *p* = 0.436).

**TABLE 3:** Daily distribution of NEWS risk categories and follow‐up status in the control and study groups (*n* = 80).

Study day	NEWS risk category/Follow‐up status	Control (*n* = 40)		Study (*n* = 40)		*χ* ^2^	MCp
		*n*	%	*n*	%		
1st operation day	*NEWS risk category*						
	Low risk	2^a^	5.0	5^a^	12.5		
	Moderate risk	34^a^	85.0	33^a^	82.5	1.892	0.436
	High risk	4^a^	10.0	2^a^	5.0		
2nd day	*NEWS risk category*						
	Low risk	3^a^	7.5	6^a^	15.0		
	Moderate risk	30^a^	75.0	30^a^	75.0	1.770	0.417
	High risk	7^a^	17.5	4^a^	10.0		
3rd day	*NEWS risk category*						
	Low risk	4^a^	10.0	14^b^	35.0		
	Moderate risk	15^a^	37.5	16^a^	40.0	9.491*	0.009*
	High risk	21^a^	52.5	10^b^	25.0		
4th day	*NEWS risk category*						
	Low risk	3^a^	7.5	8^a^	20.0		
	Moderate risk	22^a^	55.0	25^a^	62.5	5.373	0.068
	High risk	15^a^	37.5	7^b^	17.5		
5th day	*NEWS risk category*						
	Low risk	7^a^	17.5	8^a^	20.0		
	Moderate risk	20^a^	50.0	23^a^	57.5	1.027	0.621
	High risk	13^a^	32.5	9^a^	22.5		
6th day	*NEWS risk category*						
	Low risk	9^a^	22.5	10^a^	25.0		
	Moderate risk	14^a^	35.0	22^a^	55.0	5.070	0.079
	High risk	17^a^	42.5	8^b^	20.0		
7th day	NEWS risk category						
	Low risk	8^a^	20.0	12^a^	30.0		
	Moderate risk	19^a^	47.5	20^a^	50.0	2.016	0.365
	High risk	13^a^	32.5	8^a^	20.0		
8th day	*NEWS risk category*						
	Low risk	12^a^	30.0	15^a^	37.5		
	Moderate risk	13^a^	32.5	16^a^	40.0	2.144	0.342
	High risk	15^a^	37.5	9^a^	22.5		
9th day	*NEWS risk category*						
	Low risk	10^a^	25.0	15^a^	37.5		
	Moderate risk	14^a^	35.0	19^a^	47.5	6.303*	0.043*
	High risk	16^a^	40.0	6^b^	15.0		
10th day	*NEWS risk category*	*N* = 40		*N* = 35			
	Low risk	15^a^	37.5	20^a^	57.1		
	Moderate risk	14^a^	35.0	12^a^	34.3		
	High risk	11^a^	27.5	3^b^	8.6	5.129	0.077
11th day	*NEWS risk category*	*N* = 38		*N* = 23			
	Low risk	15^a^	39.5	10^a^	43.5		
	Moderate risk	17^a^	44.7	11^a^	47.8		
	High risk	6^a^	15.8	2^a^	8.7	0.636	0.728
12th day	*NEWS risk category*	*N* = 38		*N* = 19			
	Low Risk	17^a^	44.7	12^a^	63.2		
	Moderate risk	16^a^	42.1	5^a^	26.3		
	High risk	5^a^	13.2	2^a^	10.5	1.733	0.451
13th day	*NEWS risk category*	*N* = 34		*N* = 17			
	Low risk	14^a^	41.2	12^a^	70.6		
	Moderate risk	14^a^	41.2	4^a^	23.5		
	High risk	6^a^	17.6	1^a^	5.9	4.066	0.054
14th day	NEWS risk category	*N* = 27		*N* = 16			
	Low risk	11^a^	40.7	10^a^	62.5		
	Moderate risk	14^a^	51.9	5^b^	31.3		
	High risk	2^a^	7.4	1^a^	6.3	2.047	0.396
15th day	NEWS risk category	*N* = 24		*N* = 7			
	Low risk	15^a^	62.5	4^b^	57.1		
	Moderate risk	7^a^	29.2	3^a^	42.9		
	High risk	2^a^	8.3	0^a^	0.0	0.771	0.799
Fr (*p*)		16.396 (0.059)		60.637* (< 0.001*)			
*η* ^2^ (95% CI)		0.062 (−0.006–0.088)		0.112 (0.048–0.141)			

*Note:* a,b: Bonferroni correction for chi‐squared test (common letters are not significant, i.e., different letters are significant).

Abbreviations: *χ* ^2^, chi‐squared test; *η* ^2^, partial eta square; CI, confidence interval; Fr, Friedman test; MC, Monte Carlo significance (two‐sided).

* Statistically significant at *p* ≤ 0.01.

As follow‐up progressed, daily cross‐sectional comparisons evaluated strictly active patients remaining in the unit, with sample sizes (*N*) adjusted daily to account for patient discharges. On Days 3 and 9, the intervention group demonstrated significantly lower proportions of high‐risk patients compared with the control group (*p* = 0.009 and *p* = 0.043, respectively). From Days 10 through 14, statistical test metrics (chi‐square and Monte Carlo *p*‐values) reflect exclusively the risk level distribution among remaining non‐discharged patients, ensuring complete analytical isolation between NEWS categories and discharge endpoints.

Parallel to physiological stabilization, patient discharge rates steadily increased in the intervention group starting from Day 10. By Day 11, 17 patients (42.5%) in the intervention group had achieved clinical stability and were successfully discharged, compared with only 2 patients (5.0%) in the control group. This trend continued through Day 14, where 24 patients (60.0%) in the intervention group were discharged versus 13 patients (32.5%) in the control group. Overall, the intervention group demonstrated a faster transition toward physiological stability and earlier ICU/hospital discharge compared with routine care.

Table 4 presents the distribution of individual stoma‐site infection‐related signs among the control and intervention groups during follow‐up. No stoma‐related abnormalities were observed at baseline immediately after tracheostomy insertion in either group.

**TABLE 4:** Signs of stoma site infections among studied groups (*n* = 80).

Stoma‐site infection‐related signs	Time points	Control (*n* = 40)		Study (*n* = 40)		*χ* ^2^	*p*
		*n*	%	*n*	%		
Redness	Baseline (immediately after insertion)	0	0.0	0	0.0	—	—
	Follow‐up (1 week)	10^a^	25.0	5^a^	12.5	2.051	0.152
	Follow‐up (2 weeks)	14^a^	35.0	5^b^	12.5	5.591*	0.018*
	Fr (*p*)	13.0* (0.002*)		5.00 (0.082)			
	Baseline (immediately after insertion)	0	0.0	0	0.0	—	—
	Follow‐up (1 week)	15^a^	37.5	7^b^	17.5	4.013*	0.045*
	Follow‐up (2 weeks)	8^a^	20.0	3^a^	7.5	2.635	0.105
	Fr (*p*)	14.696* (0.001*)		7.400* (0.025*)			
Warmth around the stoma	Baseline (immediately after insertion)	0	0.0	0	0.0	—	—
	Follow‐up (1 week)	10^a^	25.0	6^a^	15.0	1.250	0.264
	Follow‐up (2 weeks)	10^a^	25.0	2^b^	5.0	6.275*	0.012*
	Fr (p)	10.00*(0.007*)		7.000*(0.030*)			
Purulent discharge	Baseline (immediately after insertion)	0	0.0	0	0.0	—	—
	Follow‐up (1 week)	11^a^	27.5	4^b^	10.0	4.021*	0.045*
	Follow‐up (2 weeks)	7^a^	17.5	2^a^	5.0	3.130	0.154
	Fr (p)	10.33* (0.006*)		4.000 (0.135)			
Skin breakdown or ulceration near the stoma	Baseline (immediately after insertion)	0	0.0	0	0.0	—	—
	Follow‐up (1 week)	15^a^	37.5	7^b^	17.5	4.013*	0.045*
	Follow‐up (2 weeks)	7^a^	17.5	4^a^	10.0	0.949	0.330
	Fr (p)	15.364* (0.001*)		6.727* (0.035*)			
Foul‐smelling drainage	Baseline (immediately after insertion)	0	0.0	0	0.0	—	—
	Follow‐up (1 week)	13^a^	32.5	3^b^	7.5	7.813*	0.005*
	Follow‐up (2 weeks)	7^a^	17.5	1^b^	2.5	5.00*	0.025*
	Fr (p)	12.700* (0.002*)		3.500 (0.174)			

*Note:* a,b: Bonferroni correction for chi‐squared test (common letters are not significant, i.e., different letters are significant).

Abbreviations: *χ* ^2^, chi‐squared test; Fr, Friedman test; MC, Monte Carlo significance (two‐sided); —, not applicable.

* Statistically significant at *p* ≤ 0.01.

During follow‐up, the intervention group generally demonstrated lower frequencies of stoma‐site complications compared with the control group. Significant between‐group differences were observed in several components, including redness at 2 weeks (*p* = 0.018), swelling at 1 week (*p* = 0.045), warmth around the stoma at 2 weeks (*p* = 0.012), purulent discharge at 1 week (*p* = 0.045), skin breakdown or ulceration at 1 week (*p* = 0.045), and foul‐smelling drainage at both 1 week (*p* = 0.005) and 2 weeks (*p* = 0.025).

Within‐group analysis showed significant changes over time in several stoma‐related signs among the control group, including redness, swelling, warmth, purulent discharge, skin breakdown, and foul‐smelling drainage. In contrast, the intervention group demonstrated fewer significant changes over time, suggesting better maintenance of stoma‐site condition during follow‐up.

Table 5 shows that the overall stoma‐integrity composite scores were significantly lower in the study group than in the control group at both follow‐up assessments, indicating better stoma integrity among patients who received the intervention. At 1 week, the mean composite score was 0.80 ± 1.24 in the study group compared with 1.85 ± 1.71 in the control group, with a mean between‐group difference of 1.05 (95% CI: 0.39–1.72, *p* = 0.002). At 2 weeks, the study group continued to demonstrate a significantly lower composite score than the control group (0.43 ± 0.81 vs. 1.33 ± 1.23), with a mean difference of 0.90 (95% CI: 0.44–1.36, *p* = 0.001). Within‐group analysis also demonstrated significant changes in stoma‐integrity scores over time in both the control group (Friedman *χ* ^2^ = 23.059, *p* < 0.001, Kendall's *W* = 0.378) and the study group (Friedman *χ* ^2^ = 43.593, *p* < 0.001, Kendall's *W* = 0.404).

**TABLE 5:** Overall stoma integrity composite score across follow‐up in the control and study groups.

Overall stoma‐integrity composite	Control (*n* = 40)	Study (*n* = 40)	Mean difference between‐group difference (95% CI)	*p*
Baseline (immediately after insertion)	0.0 ± 0.0	0.0 ± 0.0	—	—
Follow‐up (1 week)	1.85 ± 1.71	0.80 ± 1.24	1.05 (95% CI: 0.39–1.72)	0.002*
Follow‐up (2 weeks)	1.33 ± 1.23	0.43 ± 0.81	0.90 (95% CI: 0.44–1.36)	0.001*
Within‐group change over time				
Friedman test	23.059 (< 0.001*)	43.593 (< 0.001*)	—	—

*Note:* Mean difference represents the difference in overall stoma‐integrity composite scores between the control and intervention groups. Friedman test was used to assess within‐group changes over time.

Abbreviations: CI, confidence interval; —, not applicable.

* *p* ≤ 0.01 was considered statistically significant.

Table 6 shows primary repeated‐measures ANOVA that demonstrated a statistically significant Group × Time interaction effect for the overall stoma‐integrity composite score (𝐹 = 5.849, *p* < 0.001, *ηp* ^2^ = 0.048). Significant main effects were observed for both study group (𝐹 = 22.987, *p* < 0.001,*ηp* ^2^ = 0.089) and time (𝐹 = 32.932, *p* < 0.001, *ηp* ^2^ = 0.220). Estimated marginal means reflected lower composite impairment scores in the intervention group (0.425, 95% CI: 0.169–0.681) compared to the control group (0.900, 95% CI: 0.435–1.365).

**TABLE 6:** Longitudinal changes in stoma integrity and follow‐up status in the control and study group.

Overall stoma‐integrity composite	Estimate	95% CI	*F*	*p*	Partial eta squared (95% CI)
Group					
Control	0.425	0.169–0.681	22.987*	< 0.001*	0.089 (0.008–0.194)
Bundle	0.900	0.435–1.365			
Time	0.375	0.094–0.844	32.932*	< 0.001*	0.220 (0.141–0.393)
Group × Time	0.150	0.061–0.961	5.849*	< 0.001*	0.048 (0.007–0.125)

*Note:* Group × Time interaction represents the primary analysis to determine whether changes in the outcome over time differed between the control and intervention groups.

Abbreviations: *ηp* ^2^, partial eta squared; CI, confidence interval; *F*, *F* statistic.

* Statistically significant at *p* ≤ 0.01.

Table 7 presents at baseline, respiratory distress levels measured via RDOS were equivalent between groups, with 87.5% of control patients and 90.0% of study group patients exhibiting no distress (χ^2^ = 0.452, *p* = 1.000). At 1‐week follow‐up, a significantly higher proportion of study group patients experienced no respiratory distress compared to controls (52.5% vs. 10.0%), whereas moderate (25.0% vs. 12.5%) and severe distress (12.5% vs. 5.0%) were more prevalent in the control group (*χ* ^2^ = 17.353, *p* = 0.001). This pattern persisted at 2‐week follow‐up, with 77.5% of intervention patients demonstrating no distress compared to 47.5% of control patients (*χ* ^2^ = 7.947, *p* = 0.047). Within‐group longitudinal changes across time points were significant for both the control (Fr = 16.116, *p* < 0.001) and study groups (Fr = 35.765, *p* < 0.001).

**TABLE 7:** Respiratory distress categories (RDOS) among control and intervention groups (*n* = 80).

Respiratory distress observation scale	Control (*n* = 40)		Study (*n* = 40)		*χ* ^2^	MCp
	*n*	%	*n*	%		
Baseline (immediately after insertion)						
None	35^a^	87.5	36^a^	90.0		
Mild distress	4^a^	10.0	3^a^	7.5		
Moderate distress	1^a^	2.5	1^a^	2.5	0.452	1.000
Severe distress	0	0.0	0	0.0		
Follow‐up (1 week)						
None	4^a^	10.0	21^b^	52.5		
Mild distress	21^a^	52.5	12^b^	30.0		
Moderate distress	10^a^	25.0	5^a^	12.5	17.353*	0.001*
Severe distress	5^a^	12.5	2^a^	5.0		
Follow‐up (2 weeks)						
None	19^a^	47.5	31^b^	77.5		
Mild distress	10^a^	25.0	5^a^	12.5		
Moderate distress	7^a^	17.5	3^a^	7.5	7.947*	0.047*
Severe distress	4^a^	10.0	1^a^	2.5		
Fr (*p*)	16.116* (< 0.001*)		35.765* (< 0.001*)			

*Note:* a,b: Bonferroni correction for chi‐squared test (common letters are not significant, i.e., different letters are significant).

Abbreviations: *χ* ^2^, chi‐squared test; Fr, Friedman test; MC, Monte Carlo significance (two‐sided).

* Statistically significant at *p* ≤ 0.01.

In Table 8 Longitudinal repeated‐measures modelling confirmed a statistically significant Group × Time interaction effect for respiratory distress categories (𝐹 = 4.382, *p* = 0.014, *ηp* ^2^ = 0.036). Significant main effects were observed for both group (𝐹 = 18.901, *p* < 0.001, *ηp* ^2^ = 0.075) and time (𝐹 = 29.088, *p* < 0.001, *ηp* ^2^ = 0.199). The estimated marginal means indicated a lower mean distress category rating in the study group (0.350, 95% CI: 0.118–0.582) relative to the control group (0.550, 95% CI: 0.150–0.950), demonstrating greater longitudinal reduction in respiratory distress trajectories among intervention participants.

**TABLE 8:** Longitudinal changes respiratory distress categories in the control and study group.

Respiratory distress	Estimate	95% CI	*F*	*p*	Partial eta squared (95% CI)
Group					
Control	0.350	0.118–0.582	18.901*	< 0.001*	0.075 (0.004–0.193)
Bundle	0.550	0.150–0.950			
Time	0.350	0.084–0.405	29.088*	< 0.001*	0.199 (0.078–0.248)
Group × Time	0.150	0.013–0.713	4.382*	0.014*	0.036 (0.001–0.106)

*Note:* Group × Time interaction represents the primary analysis to determine whether changes in the outcome over time differed between the control and intervention groups.

Abbreviations: *ηp* ^2^, partial eta squared; CI, confidence interval; *F*, *F* statistic.

* Statistically significant at *p* ≤ 0.01.

Table 9 presents the distribution of CPIS‐indicated suspected respiratory infection among the control and intervention groups during follow‐up. At baseline immediately after tracheostomy insertion, no participants in either group met the criteria for CPIS‐indicated respiratory infection.

**TABLE 9:** The rate of respiratory tract infections in the studied groups using CPIS.

Days	Diagnosis of respiratory tract infections (pneumonia) using CPIS								
	Control group (*n* = 40)				Study group (*n* = 40)				*χ* ^2^/MCp
	No		Yes		No		Yes		
	*n*	%	*n*	%	*n*	%	*n*	%	
Day 1 Immediately After Insertion	40^a^	100.	0.0	0	40^a^	100	0.0	0	—
Follow‐up (1 week) Early onset pneumonia	32^a^	80	8^a^	20.0	36^a^	90	4^a^	10.0	3.710 0.168
Follow‐up (2 weeks) Late onset pneumonia	36^a^	90	4^a^	10.0	38^a^	95	2^a^	5.0	
Fr (*p*)	8.0* (0.018*)				4.00 (0.135)				

*Note:* a,b: Bonferroni correction for chi‐squared test (common letters are not significant, i.e., different letters are significant).

Abbreviations: *χ* ^2^, chi‐squared test; Fr, Friedman test; MC, Monte Carlo significance (two‐sided); —, not applicable.

* Statistically significant at *p* ≤ 0.05.

At 1 week of follow‐up, suspected early‐onset respiratory infection was observed in 20.0% of the control group and 10.0% of the intervention group; however, this difference was not statistically significant (*p* = 0.168). At 2 weeks, suspected late‐onset respiratory infection was observed in 10.0% of the control group compared with 5.0% of the intervention group, with no statistically significant difference between groups. Within‐group analysis showed significant changes over time in CPIS‐indicated infection status in the control group, whereas no statistically significant change was observed in the intervention group.

## 5 Discussion

Tracheostomy management in CIPs requires consistent airway management, stoma care, and infection prevention practices to minimize complications and support patient safety (Billington and Luckett 2019). This study evaluated the association between a 12‐element nurse‐led tracheostomy care bundle and clinical outcomes compared with routine care, including stoma‐site conditions, respiratory distress, physiological stability, and CPIS‐indicated suspected respiratory infection. Overall, the findings demonstrated more favourable trends in several clinical outcomes among patients receiving the care bundle; however, interpretation should consider the quasi‐experimental design, non‐random allocation, and the possibility of residual confounding and measurement bias.

The demographic and clinical characteristics of the two groups showed no statistically significant differences in the measured baseline variables, suggesting general comparability regarding the observed characteristics. The predominance of males and the higher proportion of patients aged 40–50 years are consistent with previous reports describing critically ill populations requiring tracheostomy (Al‐Metyazidy et al. 2025). The high prevalence of comorbidities, particularly cardiovascular disorders, reflects the complex clinical profile commonly observed among ICU patients (Suliman et al. 2024). Nevertheless, the absence of significant baseline differences should not be interpreted as complete elimination of confounding, as non‐random allocation may allow unmeasured factors to influence outcomes.

Both groups demonstrated comparable NEWS categories during the initial assessment period, indicating similar baseline physiological status. Differences between groups became more apparent during follow‐up, with a greater proportion of patients in the study group demonstrating lower‐risk NEWS categories compared with the control group. By the end of follow‐up, the study group also showed a higher proportion of discharged patients and fewer patients remaining within higher‐risk NEWS categories.

The observed improvement in NEWS trajectory may be explained by the structured and proactive nature of the tracheostomy care bundle. Regular physiological assessment, standardized nursing actions, and early identification of airway‐related problems may facilitate timely clinical responses before deterioration progresses (Peña‐López et al. 2016). In CIPs with tracheostomies, changes in respiratory status can occur rapidly due to secretion retention, airway obstruction, or ineffective tube management; therefore, systematic monitoring may contribute to improved recognition of clinical changes (Gerry et al. 2020). However, discharge status represents a separate clinical outcome and should not be considered a component of NEWS classification. Therefore, interpretation of NEWS changes was considered separately from discharge outcomes.

Regarding stoma‐site integrity, patients receiving the tracheostomy care bundle demonstrated fewer observed local complications compared with the control group, including lower occurrence of swelling, purulent discharge, skin breakdown, redness, warmth, and foul‐smelling drainage during follow‐up. These findings may reflect the potential contribution of standardized stoma assessment, consistent hygiene practices, and structured nursing interventions in reducing variation in daily tracheostomy‐site management.

A possible explanation for these findings is that standardized care bundles provide nurses with a systematic framework for identifying early local changes and applying preventive measures before complications become clinically significant. Regular inspection of the stoma site, appropriate cleaning procedures, and consistent documentation may enhance continuity of care and reduce missed early signs of inflammation or local infection (Divo 2017). Previous studies have reported that structured tracheostomy care protocols may improve adherence to recommended practices and reduce local complications by promoting standardized nursing care (Al‐Metyazidy et al. 2025; Suliman et al. 2024). However, because stoma‐site assessment included observational components and blinding was not fully feasible, assessor‐related bias should be considered when interpreting these findings.

Patients receiving the tracheostomy care bundle demonstrated lower observed respiratory distress levels based on RDOS categories after 1 and 2 weeks of follow‐up. This improvement may be related to the bundle components targeting airway patency, secretion management, humidification, and tube‐care practices, which are important factors influencing respiratory comfort and airway function.

The potential mechanism underlying improved respiratory outcomes may involve earlier recognition and management of airway‐related problems. Tracheostomy patients are particularly vulnerable to secretion accumulation, impaired clearance, and changes in respiratory effort; therefore, standardized nursing surveillance may allow earlier intervention and reduce the progression of respiratory distress (Bahig Anwr Akl et al. 2023). These findings are consistent with previous reports suggesting that structured tracheostomy management approaches may improve respiratory outcomes (Olton et al. 2019; Mohmed Mahmod Allam et al. 2024). Nevertheless, RDOS includes observational assessments; therefore, potential measurement bias should be considered, particularly within a non‐blinded study design.

The study group demonstrated lower proportions of CPIS‐indicated suspected respiratory infection compared with the control group; however, these differences did not reach statistical significance. Therefore, the findings should not be interpreted as evidence of a confirmed reduction in pneumonia incidence. The lack of statistical significance may be explained by several factors, including the relatively small sample size, limited follow‐up duration, and the absence of microbiological confirmation within the modified CPIS assessment.

Although the care bundle may influence early indicators of airway management quality and local stoma condition, infection‐related outcomes may require longer observation periods and larger sample sizes to demonstrate meaningful differences (McGrath et al. 2020). Respiratory infections among tracheostomy patients are influenced by multiple factors, including baseline illness severity, duration of mechanical ventilation, colonization status, and underlying disease conditions (Singh et al. 2000), which may not be fully modified by nursing interventions alone. Previous studies have reported benefits of care bundles in reducing respiratory infections; however, differences in patient populations, bundle components, diagnostic criteria, and study designs should be considered when comparing findings (Xiao et al. 2021; Suliman et al. 2023).

Collectively, the findings suggest that a structured nurse‐led tracheostomy care bundle may have greater observable effects on outcomes directly related to daily nursing practices, such as stoma‐site management, respiratory monitoring, and early physiological assessment, compared with outcomes requiring longer‐term biological changes, such as infection development. This highlights the potential clinical relevance of standardized tracheostomy care protocols as a framework for improving consistency of nursing practice and enhancing patient monitoring in critical care settings.

Future studies should include larger multicentre samples, longer follow‐up periods, microbiological assessment of respiratory infections, and randomized or controlled designs to further evaluate the effectiveness of standardized tracheostomy care bundles and determine their independent contribution to patient outcomes.

## 6 Limitations

Several methodological, operational, and analytical limitations should be considered when interpreting these findings. Regarding study design and internal validity, the quasi‐experimental design and non‐random allocation introduce potential selection bias. Although measured baseline demographic and clinical characteristics were comparable between groups, unmeasured confounding cannot be completely excluded. Furthermore, blinding of bedside nurses was unfeasible due to the nature of the complex nursing intervention. Consequently, observational endpoints including stoma‐site evaluations and RDOS scoring remain susceptible to detection bias, despite the application of standardized objective criteria. Additionally, participant replacement following ICU transfer or early mortality during the recruitment phase may have altered sample composition, potentially introducing survivor bias into the final analytical cohort.

In terms of intervention implementation and generalizability, intervention fidelity was maintained through real‐time clinical supervision and daily oversight; however, it was not quantitatively recorded as a numerical adherence percentage. As a result, the precise dose–response relationship and individual compliance rates across all 12 bundle elements could not be quantified. Moreover, because the intervention was researcher‐supervised within a single‐centre setting using consecutive recruitment from a localized ICU population, the external validity and generalizability of the findings to broader healthcare systems may be constrained.

Finally, regarding analytical scope and long‐term endpoints, while the revised statistical framework incorporated primary longitudinal models (Group × Time interactions) to evaluate recovery trajectories, multivariable adjusted analyses for potential baseline confounders were not performed due to sample size constraints. Lastly, the follow‐up duration was restricted to 15 days, which precluded the evaluation of extended clinical outcomes such as successful decannulation rates, late structural airway complications, or long‐term infection dynamics. Future research utilizing randomized allocation, blinded outcome assessors, quantitative fidelity tracking, and comprehensive multivariable adjustments across larger multicentre cohorts is warranted to confirm and extend these results.

## 7 Recommendations and Implications for Further Research

The findings suggest that a structured nurse‐led tracheostomy care bundle may improve several aspects of tracheostomy management, particularly stoma‐site condition, respiratory distress, and physiological monitoring. However, due to the non‐randomized design, the bundle should be considered as a promising approach requiring further validation rather than established standard practice.

At the study setting, the bundle may be incorporated within a structured quality‐improvement framework through standardized protocols, bedside checklists, documentation tools, and competency‐based nurse training. Regular monitoring of bundle adherence and clinical outcomes is recommended to support continuous improvement.

At the healthcare‐system level, standardized tracheostomy care pathways may reduce variation in practice, improve multidisciplinary communication, and enhance patient safety. Implementation should consider institutional resources, staffing capacity, and patient characteristics.

Future research should include larger multicentre studies with randomized or controlled allocation methods, blinded outcome assessment, and appropriate adjustment for confounding variables. Longer follow‐up periods, objective assessment of intervention fidelity, microbiological confirmation of respiratory infections, and evaluation of outcomes such as decannulation success, healthcare utilization, and cost‐effectiveness are recommended.

## 8 Conclusion

This study evaluated the association between a 12‐element nurse‐led tracheostomy care bundle and clinical outcomes among CIPs requiring tracheostomy. The findings demonstrated that patients receiving the care bundle showed more favourable outcomes related to stoma‐site integrity, respiratory distress, and physiological stability compared with routine care. The intervention group experienced fewer observed stoma‐site complications, lower respiratory distress levels during follow‐up, and more favourable changes in NEWS‐based physiological status over time. These findings suggest that a structured nurse‐led approach may enhance the consistency of tracheostomy‐related care practices, support early recognition of complications, and improve monitoring of CIPs with tracheostomies.

However, the bundle was not associated with a statistically significant reduction in CPIS‐indicated suspected respiratory infection. This finding indicates that infection‐related outcomes may require larger sample sizes, longer observation periods, and more specific diagnostic approaches, including microbiological assessment, to determine whether standardized nursing interventions influence respiratory infection risk.

Considering the non‐randomized quasi‐experimental design, the results should be interpreted as associations rather than definitive evidence of causality. Nevertheless, the findings support the potential value of structured nurse‐led tracheostomy care bundles as part of ICU quality‐improvement strategies. Further controlled studies with rigorous methodology are required before establishing definitive recommendations for widespread routine implementation.

## Author Contributions

**Eman Yasser Hammouda:** conceptualization, supervision, methodology, writing – review and editing. **Mohammed Elsayed Zaky:** software, validation, writing – original draft, visualization. **Mona Saad Abdelwareth Ahmed:** resources, funding acquisition, project administration. **Walaa El‐Khanany Zahran:** methodology, validation, visualization, software. **Hend El‐Sayed Mansour:** data curation, methodology, conceptualization, writing – review and editing.

## Funding

This research was funded by Princess Nourah bint Abdulrahman University Researchers Supporting Project number (PNURSP2026R840), Princess Nourah bint Abdulrahman University, Riyadh, Saudi Arabia.

## Disclosure

AI Declaration: The authors confirm that NO Artificial Intelligence (AI) or AI‐assisted technologies were used to generate the research data, perform the statistical analyses, or formulate the scientific conclusions presented in this study. AI tools were utilized solely to improve the readability and linguistic phrasing of the manuscript's plain English summary and supporting documentation to ensure compliance with journal formatting requirements. The authors maintain full responsibility for the integrity, accuracy, and original content of the work.

## Ethics Statement

Ethical approval was obtained from the Research Ethics Committee of the Faculty of Nursing at Mansoura University (P.0631) Formal acceptance was also obtained from the hospital's administrative authority. After providing details about the nature of the study, including the aim, procedure, benefits, and risks, the study samples (the patients' relatives) gave written informed consent.

## Conflicts of Interest

The authors declare no conflicts of interest.

## Data Availability

Data is available from the corresponding author upon reasonable request.
